# Depressive-Like Behaviors Induced by Chronic Social Defeat Stress Are Associated With HDAC7 Reduction in the Nucleus Accumbens

**DOI:** 10.3389/fpsyt.2020.586904

**Published:** 2021-01-26

**Authors:** Weijun Qian, Chao Yu, Shuai Wang, Aijun Niu, Guangyan Shi, Yuancui Cheng, Ning Xu, Qiangqiang Jin, Xu Jing

**Affiliations:** ^1^Imaging Department, Kaifeng Central Hospital, Kaifeng, China; ^2^Department of Clinical Laboratory, The Second Hospital, Cheeloo College of Medicine, Shandong University, Jinan, China; ^3^Key Laboratory of Brain Functional Remodeling, Department of Neurosurgery, Qilu Hospital of Shandong University and Brain Science Research Institute, Shandong University, Jinan, China; ^4^Department of Obstetrics, The Second Hospital of Shandong University, Jinan, China

**Keywords:** chronic social defeat stress, depression, HDAC7, nucleus accumbens (NAc), epigenetic

## Abstract

Persistent symptoms of depression indicate the adaptive involvement of stable molecules in the brain that may be manifested at the level of chromatin remodeling, such as histone acetylation. Former studies have identified alterations in histone acetylation and deacetylation in several animal models about depression. However, the specific histone deacetylases related with depression are needed to be explored. Here, social avoidance behaviors, anxiety-, and depression-like behaviors were all found in mice suffered from chronic social defeat stress. Moreover, we also discovered that the amount of the class II histone deacetylase, HDAC7 rather than HDAC2, was significantly decreased in the nucleus accumbens of defeated mice, which suggested that HDAC7 might be a crucial histone deacetylase in a chronic social defeat stress model. Our data showed that the depressive-like behaviors induced by chronic social defeat stress were associated with HDAC7 reduction in nucleus accumbens. HDAC7 might be a promising therapeutic target for depression.

## Summary Box

**What is already known?** Persistent symptoms of depression may be manifested as changes in chromatin remodeling.
**What are the new findings?** Our data show that the depressive-like behaviors induced by chronic social defeat stress were associated with HDAC7 reduction in nucleus accumbens.
**What do the new findings imply?** HDAC7 may be a promising therapeutic target for depression.

## Introduction

According to a global report published by WHO in 2017, ~322 million people worldwide suffer from depression, of which nearly half live in the South-East Asian and Western Pacific regions (1). Depression is believed to have a close correlation with genetic material involving aberrant changes in gene expression (2, 3). Accumulating evidence has indicated that histone acetylases and deacetylases dynamically regulate epigenetic mechanisms, such as histone acetylation (4, 5). In a rodent model of depression caused by chronic social defeat stress, depressive symptoms are improved by the inhibitors of class II histone deacetylase, which will contribute to changes in gene expression related to depression similar to fluoxetine (6), thereby suggesting that class II HDACs may be a promising therapeutic target for depression.

In epigenetic mechanisms that may be associated with psychiatric illness, one of the most appealing features is changes of chromosomal structures and expressional alterations of specific genes under environmental stimuli (7, 8). Histone deacetylases (HDACs) are one type of chromatin-modifying enzymes used for the elaborate process of epigenetic regulation, therefore they could effectively link environmental stimuli with alteration of gene expression (9). HDACs are comprised of four classes, of which class I, class II, and class III are distinct based on their homology with yeast genes *rpd3, hda1*, and *sir2*, respectively; class IV includes only HDAC11 (10). HDAC inhibitor infusion into the nucleus accumbens (NAc), hippocampus, and cortex can elicit antidepressant-like responses, which supports the functional role and potential utility of such inhibitors in the treatment of depression (11–13). Moreover, treatment of mice with the inhibitor of class I and II HDAC (MS-275) reverses the effects of chronic social defeat stress on gene expression in the NAc, with a striking similarity to the function of the standard antidepressant, fluoxetine (14, 15). Despite the antidepressant effect of MS275 being confirmed, there is no direct evidence regarding which kind of HDACs as a form of essential mediators plays an important part in the anti-depressant process (16).

NAc has been demonstrated to be involved in the development of depressive disorders and the regulation of antidepressant action (17, 18). In particular, elevated levels of brain-derived neurotrophic factor (BDNF) in the NAc are associated with depressive behaviors, in which BDNF exerts an antidepressant role in the hippocampus (19). Moreover, when inhibitors of HDAC are directly infused into several brain regions including the medial prefrontal cortex, ventral hippocampus, NAc, or amygdala, they produce an antidepressant effect (20, 21).

HDAC7, an essential member of class II HDACs, is widely expressed in the central nervous system (22). Limited studies have been conducted on the function of HDAC7 in depression. Therefore, we investigated whether HDAC7 in the NAc is involved in the occurrence of depressive-like behaviors. Here, we specifically focused on the altered expression of HDAC7 in the NAc of mice who suffered from chronic social defeat stress.

## Methods

### Animals

Male C57BL/6J mice aged 6–8 weeks weighing 23–25 g and male CD-1 retired mice aged 8–9 months were purchased from Charles River (Wilmington, MA). All mice were housed in standard cages at controlled temperature (22 ± 2°C) under diurnal conditions (12 h light/dark cycle). Food and water were available *ad libitum*. All handling procedures for animals were conducted following the guidelines of the National Institutes of Health Guide for the Care and Use of Laboratory Animals and were approved by the institutional animal care and use committee of Shandong University.

### Chronic Social Defeat Stress

Chronic social defeat stress was induced as previously described (23) with modifications according to the experimental requirements. C57BL/6J mice were continuously subjected to social defeat stress for 10 days. CD1 retired breeders were used as aggressors in the experiment if they met the following criteria: Firstly, the CD1 resident aggressor must attack in at least two consecutive sessions during three consecutive screening sessions lasting for 180 s; Secondly, the attack latency recorded for each session must be <60 s. Each mouse was introduced into the homecage of a stranger CD1 resident aggressor every day and was defeated physically for 10 min. After 10 min of physical interaction, the residents and intruders were separated by perforated plexiglass to allow sensory contact in the following duration of 24 h. Every day, each mouse was introduced into a new homecage of a resident CD1 aggressor. The animals in the control group were housed in pairs on each side of a perforated plexiglass partition and were handled in the same manner as those in the experimental groups every day.

### Behavioral Procedures

Fourteen mice were subjected to social approach-avoidance test, open field test, elevated plus maze (EPM) test, tail suspension test, and forced swimming test according to the following procedures. After 5 days of the behavioral tests, mice were killed after the behavioral tests, and brain tissues were collected for western blot assay.

#### Open Field Test

An open field test was conducted as a previously described report from our laboratory with modifications (24). The spontaneous motivation ability and anxiety-like behavior were assessed in the open field test. The area of tan open field test consisted of a 40 cm × 40 cm area divided into central (20 cm × 20 cm) and peripheral regions by black metal walls with height at 35 cm. Mice could acclimatize themselves to the test room for 1 h before the experiment. During the test, mice were placed at the center region of the field, and their behaviors were recorded for 10 min. A video-tracking system (Smart 3.0, Harvard apparatus, Holliston, MA, USA) was used to score the traveled distance between the central and peripheral areas for the assessment of spontaneous locomotor activity. The number of entries to the central zone, latency to leave the central zone, and time stayed in the central zone were all recorded to evaluate anxiety-like behaviors.

#### EPM Test

As previously described (25) with modifications, the EPM was comprised of two open arms (30 cm × 5 cm) with a small raised lip (0.5 cm), two enclosed arms (30 cm × 5 cm), and a central platform (5 cm × 5 cm) at 38.5 cm above the ground. During a 5-min session, each mouse was initially placed on the center platform and facing an open arm. The time stayed in open arms and the number of entries into the open arms was scored using a video-tracking system (Smart 3.0).

#### Social Approach-Avoidance Test

The approach-avoidance behaviors of experimental mice toward an unfamiliar social target (CD1 mouse) were carried out as previously described (26) with modifications. The arena was a black metal open field (40 cm × 40 cm) placed in complete darkness. A camera equipped with an infrared filter and lights was used to perform the video recording. Each experimental mouse was introduced into the open field and its trajectory was continuously recorded for two 2.5-min sessions with a 30-s duration for the mouse to rest in its home cage. In the first session (“no target”), an empty mesh cage made of plastic (10 cm × 6.5 cm) without a target was located at one end of the open field. During the second session (“target”), an unfamiliar CD1 male mouse was introduced into the mesh cage as a social target animal with other same conditions. Between the two sessions, the experimental mouse was removed from the arena and was placed back into its home cage for ~1 min. The video-tracking data from both the “no target” and “target” conditions were used to determine the total distance moved. The time spent by the experimental mouse in the “interaction zone” (a corridor with a width of 8 cm surrounding the mesh cage) and the “corners” (two square areas opposite to the location of the target cage) was recorded using a video-tracking system (Smart 3.0).

#### Forced Swimming Test

A forced swimming test was performed as previously described in a published study from our laboratory (27). Briefly, C57BL/6J mice were placed into a glass cylinder (25 cm in height, 10 cm in diameter) filled with 22°C water up to a height of 18 cm. The percentage of time spent in an immobile state was determined in a testing period of 6 min. Immobility was defined as the absence of struggling, immobile posture, and floating on the water. Swimming was defined as intensive use of the forepaws for moving forward to the center or along the sides of the cylinder. The body was usually oriented parallel to the sides of the cylinder. Climbing was defined as active pawing of the cylinder wall and lifting of the paws above the water surface. The body was oriented with the head toward the wall, which faced perpendicularly to the side of the cylinder.

#### Tail Suspension Test

The tail-suspension test is a mouse behavioral test useful in assessing antidepressant-like activity (28). As previously described (29) with modifications, mice were suspended upside down, and a metal bar was used to tape their tails at the position of 1 cm from the tip. Their heads were held 35 cm above the ground, and a camera was used to record immobility inthe active period of 6 min. Immobility was defined as no movement of the limbs and tails. Mice that climbed up their tails were excluded from the experiment.

### Brain Tissue Collection

After 5 days of behavioral experiments, the brain, including NAc, cerebral cortex, and dorsal hippocampus were dissected, frozen, and sliced using the freezing microtome (Braintree Scientific, Braintree, MA). Brain slices were stored at −80°C until use.

### Western Blot Assay

The extraction of tissue protein from NAc was performed as previously described (21, 22). A bullet Blender Homogenizer (Nextadvance) was used to homogenize dissected brain tissue in ice-cold RIPA buffer (50 mM Tris, 150 mM NaCl, 0.1% SDS, 0.5% sodium deoxycholate, 1% IGEPAL CA-630, pH 7.4) supplemented with protease inhibitors (Complete, Roche). Extracts were centrifuged at 14,000 g for 20 min at 4°C, and then cellular proteins were collected. The concentration of total cellular proteins was determined by a standard BCA assay before added in a loading buffer at 100°C for 5 min. Then, 80 μg of lysate proteins was separated by SDS-PAGE gels and then transferred to a polyvinylidene fluoride (PVDF) membrane (Roche, Swiss). Anti-HDAC1 (Sigma, 1:1,000), anti-HDAC2 (Sigma, 1:1,000), anti-HDAC6 (Sigma, 1:1,000), anti-HDAC7 (Sigma, 1:1,000), and anti-β actin from rabbit were used as primary antibodies. Horseradish peroxidase (HRP)-conjugated goat anti-rabbit secondary antibodies (Calbiochem, 1:1,000) were used to label the rabbit-derived primary antibodies. The images of protein bands were visualized by enhanced chemiluminescence (ECL, Pierce). Densitometry analysis of the bands was performed using Quantity One (version 4.6.2, Bio-Rad).

### RNA Extraction and Quantitative PCR (qPCR)

Total RNA was extracted from NAc, cerebral cortex, and dorsal hippocampus using TRIzol-A^+^ RNA isolation reagent (TIANGEN) according to the manufacturer's protocol. cDNA was then synthesized using the ReverTra Ace qPCR RT Kit (catalog #FSQ-101; TOYOBO). Afterward, real-time qPCR was performed using an SYBR Green PCR kit (Applied Biosystem, USA). The primers were as follows: HDAC7, 5′-GCCTCCATCGACCACTTAACC-3′ (forward) and 5′-CGAGGGTATCTGTCGCAGTC-3′ (reverse); and β-actin, 5′-CGTTGACATCCGTAAAGACCTC-3′ (forward) and 5′-CCACCGATCCACACAGAGTAC-3′ (reverse). β-actin was used as an internal control. The relative expression of HDAC7 mRNA was calculated using the 2^−ΔΔCT^ method as previously described (30).

### Statistical Analysis

All data were collected from at least three independent experiments and presented as mean ± S.E.M. Comparisons of data from various behavioral tests and western blot assay between defeated and control groups were evaluated via two independent samples *t*-test. Data among multiple groups were analyzed using one-way analysis of variance (ANOVA) with the least significant difference test for pairwise comparison. A *P*-value of 0.05 was considered to be the critical cutoff value of statistical significance. All data analyses were carried out using the SPSS statistical program version 18.0 (SPSS Inc., Chicago, IL, USA).

## Results

### Chronic Social Defeat Stress Successfully Induced Social Avoidance Behaviors

**Figure 1:**
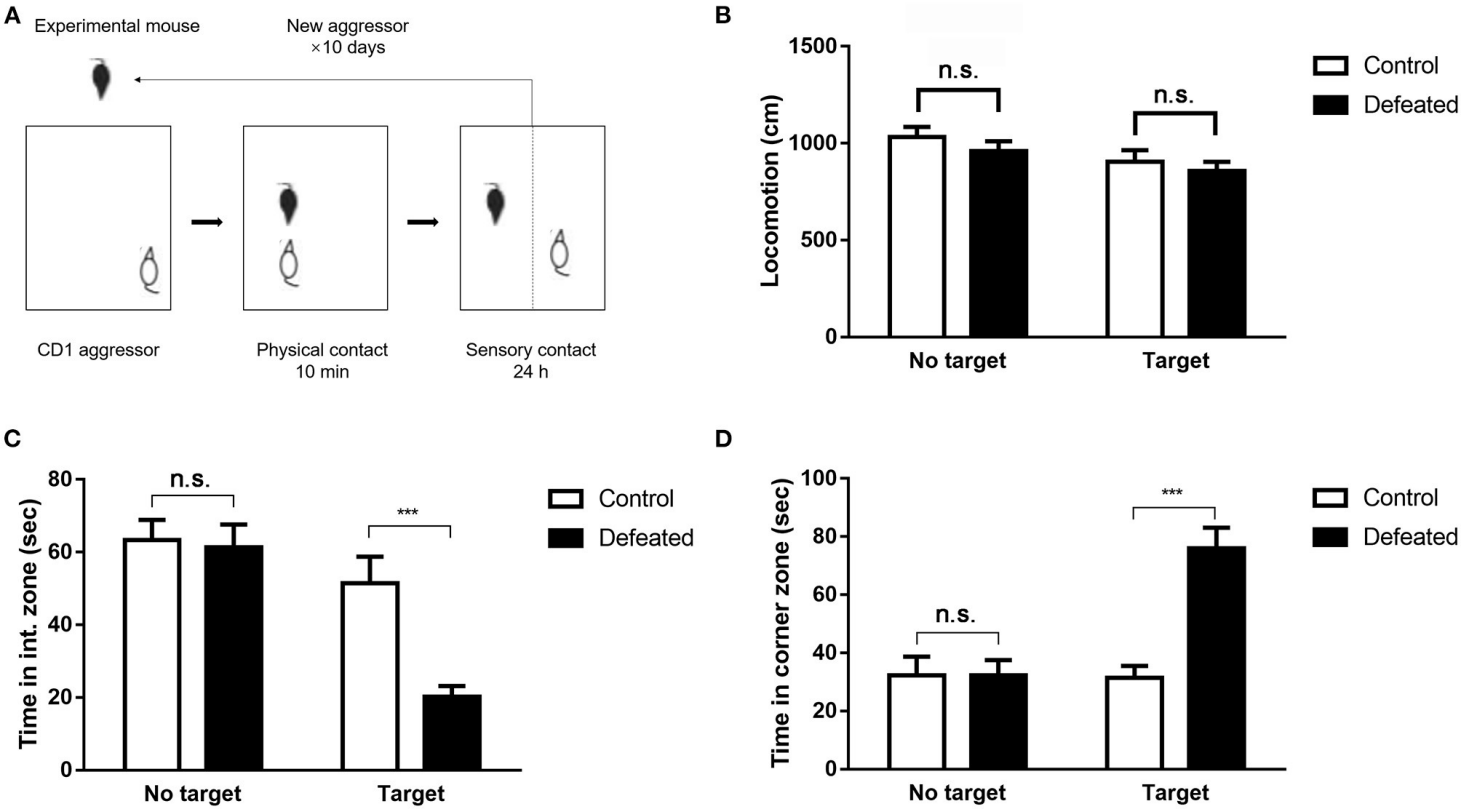
Chronic social defeat stress-induced persistent social aversion in mice. **(A)** The paradigm of chronic social defeat stress. **(B–D)** A social target decreased the time spent in the interaction zone compared with that in the control mice, and increased the time spent in the corner zone after social defeat. Chronic social defeat stress did not affect total locomotion of the experimental mice. Data are expressed as means ± S.E.M (*n* = 14 per group). Data among multiple groups were analyzed using one-way analysis of variance (ANOVA) with the least significant difference test for pairwise comparison. ***P < 0.001 compared to control.

The social approach of experimental mice toward an unfamiliar social target (CD1 mouse) enclosed in a plastic mesh cage was monitored by a video-tracking system (Figure 1A). As expected, undefeated control mice spent the most time in the interaction zone and little time in the corner zone when an unfamiliar target mouse was presented. However, defeated mice intensely displayed aversive responses to the target mouse, which spent less time in the interaction zone (*F* = 12.086, degree = 55, *P* < 0.001, Figure 1C) and preferred to staying in the corner zone (*F* = 14.017, degree = 55, *P* < 0.001, Figure 1D). This difference was observed exclusively in the presence of a social target and was not significant in an empty wire cage. No difference was observed in total movement throughout the arena (*F* = 2.124, degree = 53, *P* = 0.109, Figure 1B).

### Anxiety-Like Behavior Test

**Figure 2:**
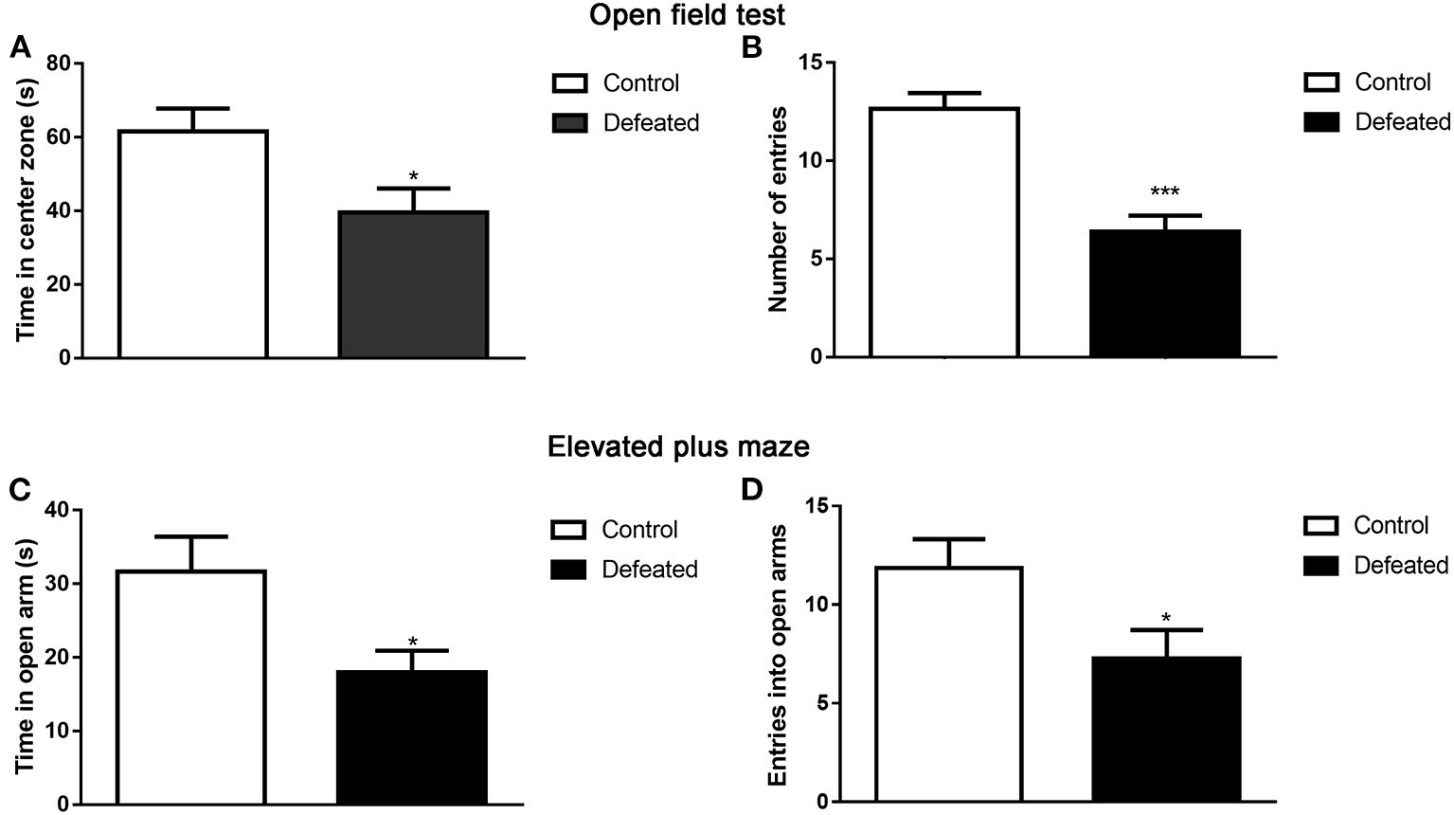
Effect of chronic social failure on anxiety-like behavior. **(A)** Compared with control mice, mice subjected to chronic social failure stress spent less time in the middle area of the open field. **(B)** Mice subjected to chronic social failure stress entered the central region less often. **(C)** Compared with control mice, mice with chronic social failure stress spent less time on the elevated cross arm. **(D)** Mice subjected to chronic social failure stress spent significantly fewer times on the open arm. Data are expressed as means ± S.E.M. (*n* = 14 per group). Data comparisons between defeated and control groups were evaluated via two independent samples *t*-test. *P < 0.05, and ***P < 0.001 compared to control.

The open-field test was performed to assess whether defeated mice displayed altered anxiety-like behaviors. Compared with that in the control group, defeated mice spent less time in the center zone of the open field (*t* = 2.445, df = 25, *P* = 0.022, Figure 2A). Furthermore, defeated mice traveled within shorter distances in the central zone and even was found to scarcely enter into the center zone (*t* = 5.425, df = 25, *P* < 0.001, Figure 2B). To further confirm the anxiety-like behaviors inflicted by chronic social defeat stress, an EPM test was also conducted. The phenomenon suggested that, relative to the control group, defeated mice significantly traveled within shorter distances (*t* = 2.444, df = 24, *P* = 0.022, Figure 2C) and less entries into the open arms (*t* = 2.239, df = 26, *P* = 0.034, Figure 2D).

### Depressive-Like Behavior Test

**Figure 3:**
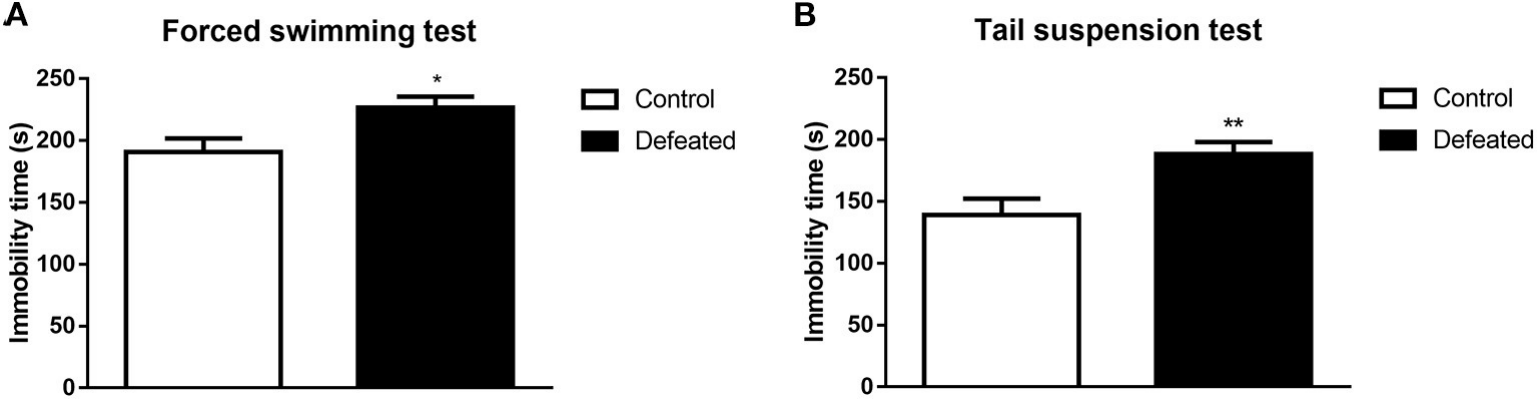
Effects of chronic social failure on depression-like behavior. **(A)** Mice subjected to chronic social failure stress were significantly more sedentary during forced swimming than control mice. **(B)** Compared with control mice, mice subjected to chronic social failure stress spent significantly more time resting in the tail suspension test. Data are expressed as means ± S.E.M (*n* = 14 per group). Data comparisons between defeated and control groups were evaluated via two independent samples *t*-test. *P < 0.05, and **P < 0.01 compared to control.

To evaluate the depressive-like behavioral changes in mice suffered from chronic social defeat stress, forced swimming, and tail suspension tests were conducted in sequence. As expected, mice defeated by aggressors displayed increased immobility time during the forced swimming test (*t* = −2.534, df = 26, *P* = 0.018, Figure 3A). To further confirm our results, we conducted a tail suspension test, where the immobility time of mice subjected to chronic social defeat stress was also increased during tail suspension (*t* = −2.979, df = 26, *P* = 0.006, Figure 3B).

### Chronic Social Defeat Stress Represses HDAC7 Expression in the NAc

**Figure 4:**
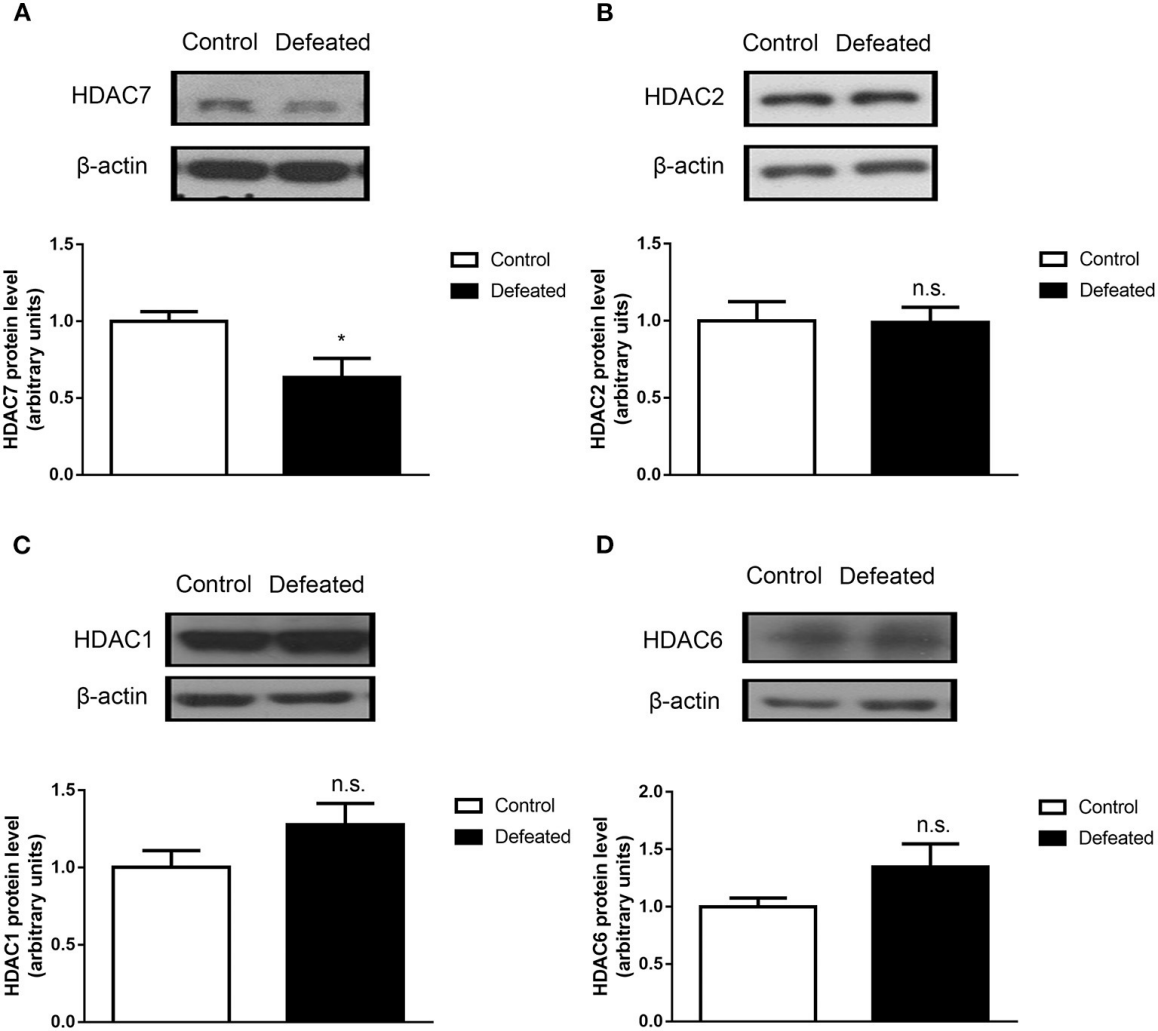
Chronic social stress failure significantly reduced HDAC7 protein expression in the nucleus accumbens. **(A)** Compared with control mice, HDAC7 protein expression in the nucleus accumbens region in the brain of mice subjected to chronic social failure stress was significantly decreased. **(B–D)** There was no significant change in the expression of HDAC1, HDAC2, and HDAC6 in the nucleus accumbens region in the brains of mice subjected to chronic social failure stress compared to those in the control group. Data are expressed as means ± S.E.M. (*n* ≥ 4 per group). Data comparisons between defeated and control groups were evaluated via two independent samples *t*-test. *P < 0.05 compared to control.

**Figure 5:**
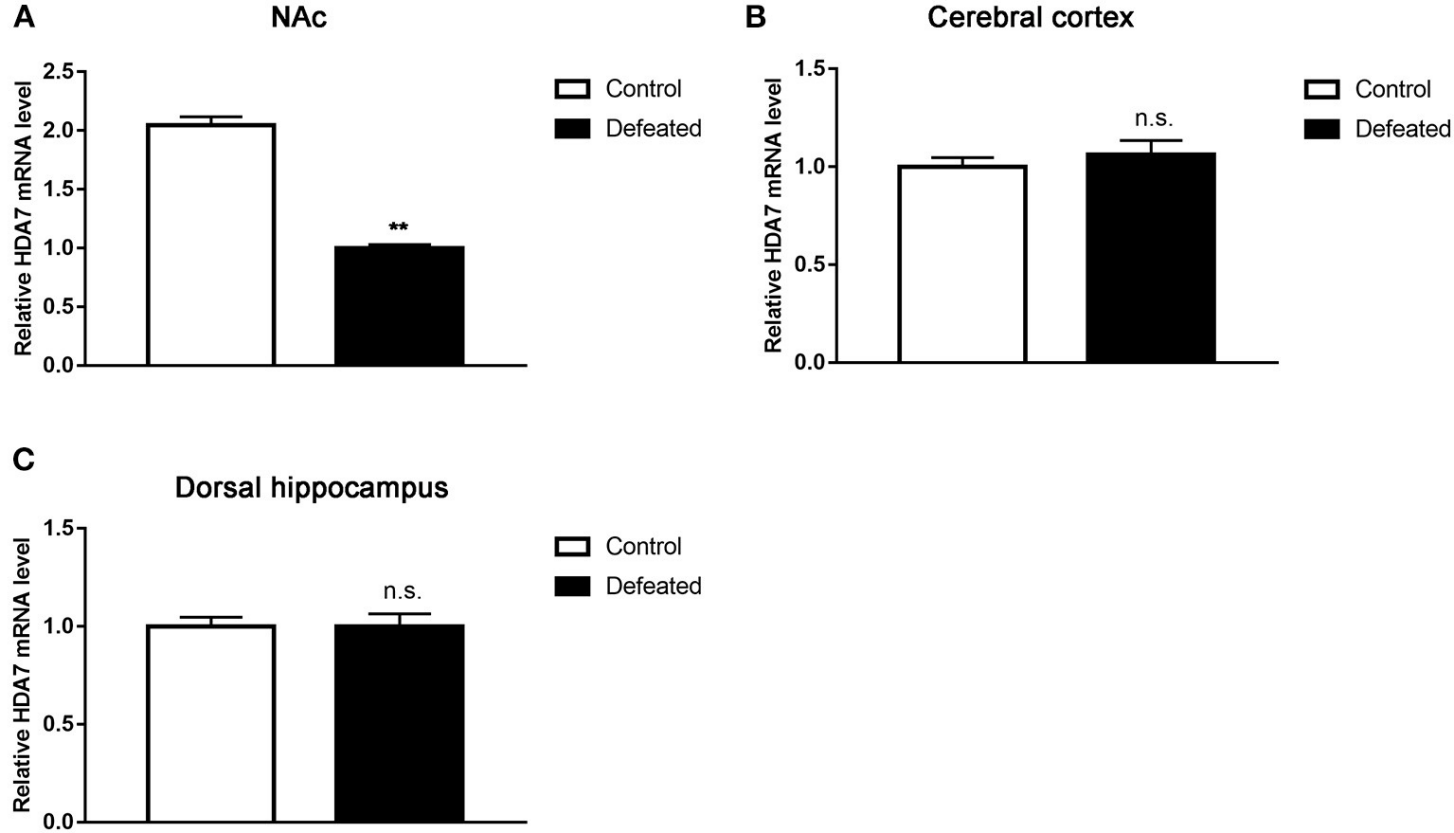
Chronic social stress failure significantly reduced HDAC7 mRNA expression in the nucleus accumbens **(A)** rather than in the cerebral cortex **(B)** and dorsal hippocampus **(C)**. Data are expressed as means ± S.E.M. (*n* ≥ 4 per group). Data comparisons between defeated and control groups were evaluated via two independent samples *t*-test. **P < 0.01 compared to control.

To analyze whether HDACs contributed to depression caused by chronic social defeat stress, western blot assay was adopted. The results showed that HDAC7 protein expression was significantly decreased in the NAc in the brain of mice subjected to chronic social failure stress compared to that in control mice (*t* = 2.614, df = 14, *P* = 0.020, Figure 4A). However, other HDACs such as HDAC1 (*t* = 0.057, df = 20, *P* = 0.955, Figure 4B), HDAC2 (*t* = −1.595, df = 7, *P* = 0.155, Figure 4C), and HDAC6 (*t* = −1.781, df = 7, *P* = 0.118, Figure 4D) were not changes in the NAc. Strikingly, it was observed that HDAC7 mRNA was also significantly decreased in the NAc (*t* = −22.588, df = 4, *P* < 0.001, Figure 5A) rather than in the cerebral cortex (*t* = −1.229, df = 4, *P* = 0.286, Figure 5B) and dorsal hippocampus (*t* = 0.007, df = 4, *P* = 0.995, Figure 5C) under chronic social defeat stress, indicating a brain region-specific role of HDAC7 in behavior changes inflicted by chronic social defeat stress. These data suggested that HDAC7 reduction in the NAc might be associated with behavior changes inflicted by chronic social defeat stress.

## Discussion

Recently, accumulated evidence have indicated that alteration of histone acetylation caused by abnormal expression of HDACs in the NAc plays an important role in depression, and overexpression of specific HDACs in the NAc can exert therapeutic effects in depressive mice (18). Here, we implemented a chronic social defeat stress mouse model for depression, in which mice were continuously defeated for 10 min by a new CD-1 aggressor every day for 10 days. Social approach-avoidance test, open field test, EPM, and forced swimming test were used to evaluate the changes in behaviors induced by the depression model. The levels of social avoidance, depression, and anxiety in the defeated mice were significantly higher than those in normal mice. Studies based on models about chronic social defeat stress obtained comparable results in social avoidance behavior, anxiety-like behavior, and depressive-like behaviors, thus corroborating our findings (23). These results suggested that mice were suitable for exploring depression- and anti-depressive response-related cellular and molecular mechanisms.

The NAc is implicated in depression, and abnormal expression of HDACs such as HDAC5 and HDAC2 has been observed in the NAc (31, 32). However, as an essential member of class II HDACs, the function of HDAC7 in depression is unclear. Our previous work indicated that HDAC7 was ubiquitously expressed in the brain including the PFC, hippocampus, and amygdala (22). The results presented here showed that the level of HDAC7 was remarkably decreased in the NAc under chronic social defeat stress.

The strengths of our study were that a well-validated mouse model of depression and several complementary behavioral assays were used to confirm that social defeat stress generated a pattern of depressive-like and anxiety-like behaviors in mice. Moreover, we found that chronic social defeat stress in mice resulted in the reduction in HDAC7 levels in the NAc, which provided a new insight for the potential mechanism involved in depression. Despite the clear strength of our study, some limitations merit further consideration. Firstly, we only observed that chronic social defeat stress represses HDAC7 expression in the NAc, whether HDAC inhibition resulted in antidepressant-like activity and some mood-stabilizing drugs (such as valproate) had HDAC inhibition properties were largely unknown. Secondly, the genes regulated by HDAC7 during chronic social defeat stress were not investigated. Exploration of manipulating HDAC7 on patterns of gene expression will facilitate to provide new insight into the possible mechanisms underlying depression. Thirdly, it is revealed that detecting the role of manipulating HDAC5 in the behavioral adaptations to chronic emotional stimuli provides strong evidence to support the effects of HDAC5 in the pathogenesis of drug addiction, depression, and other stress-related syndromes (33). Future studies elucidating the role of knockdown or overexpression of HDAC7 in NAc in regulating the behavioral adaptations to chronic social defeat stress are required. Lastly, investigation of the molecular basis of susceptibility and resistance to social defeat stress in brain reward regions helps to better maintain emotional homeostasis (34). Therefore, it is worth investigating the role of susceptibility and resilience to 10 days of social defeat stress. Observation of any degree of variation in social interaction scores that may be correlated with HDAC7 expression in NAc will confirm the function of HDAC7 in the regulation of depression.

Notably, only male mice were used in this study. The reason is to avoid the interference of the female estrus cycle. The monthly changes in estrogen and progesterone are significant in females, and these changes can affect the response state of the tested animals to certain experimental factors. Since female humans have higher rates of depression than male humans, it is unclear whether studies using only male mice have translational promise at all for understanding depression. More studies are still required to confirm our findings.

## Conclusion

In summary, our study demonstrated that HDAC7 was a critical histone deacetylase, whose reduction might be associated with the depressive-like behaviors induced by chronic social defeat stress. Therefore, HDAC7 may be a promising therapeutic target for curing depression. Our findings will provide a new insight for elucidating the mechanism underlying depression.

## Data Availability Statement

The raw data supporting the conclusions of this article will be made available by the authors, without undue reservation.

## Ethics Statement

The animal study was reviewed and approved by all handling procedures for animals were conducted following the guidelines of the National Institutes of Health Guide for the Care and Use of Laboratory Animals, and were approved by the institutional animal care and use committee of Shandong University.

## Author Contributions

All authors listed have made a substantial, direct and intellectual contribution to the work, and approved it for publication.

## Conflict of Interest

The authors declare that the research was conducted in the absence of any commercial or financial relationships that could be construed as a potential conflict of interest.
